# Proportion of Antipsychotics with CYP2D6 Pharmacogenetic (PGx) Associations Prescribed in an Early Intervention in Psychosis (EIP) Cohort: A Cross-Sectional Study

**DOI:** 10.1177/02698811241238283

**Published:** 2024-03-17

**Authors:** Adam Jameson, Muhammad Faisal, Beth Fylan, Greg C Bristow, Jaspreet Sohal, Caroline Dalton, Gurdeep S Sagoo, Alastair G Cardno, Samantha L McLean

**Affiliations:** 1 Bradford District Care NHS Foundation Trust, Bradford, UK; 2 School of Pharmacy & Medical Sciences, University of Bradford, Bradford, UK; 3 Wolfson Centre for Applied Health Research, Bradford, UK; 4 Faculty of Health Studies, University of Bradford, Bradford, UK; 5 NIHR Yorkshire and Humber Patient Safety Research Collaboration (YH PSRC), Bradford, UK; 6 Biomolecular Sciences Research Centre, Sheffield Hallam University, Sheffield, UK; 7 Population Health Sciences Institute, Newcastle University, Newcastle upon Tyne, UK; 8 Leeds Institute of Health Sciences, Faculty of Medicine and Health, University of Leeds, Leeds, UK

**Keywords:** Pharmacogenomics, Pharmacogenetics, Personalised Medicine, Psychosis, Schizophrenia, Antipsychotics, Receptor Antagonist (D2), Receptor Antagonist (D2, 5-HT2), Receptor Partial Agonist (D2, 5-HT1A), Receptor Antagonist (D2, 5-HT2, NE, alpha-2)

## Abstract

**Background:**

Prescribing drugs for psychosis (antipsychotics) is challenging due to high rates of poor treatment outcomes, which are in part explained by an individual’s genetics. Pharmacogenomic (PGx) testing can help clinicians tailor the choice or dose of psychosis drugs to an individual’s genetics, particularly psychosis drugs with known variable response due to CYP2D6 gene variants (‘CYP2D6-PGx antipsychotics’).

**Aims:**

This study aims to investigate differences between demographic groups prescribed ‘CYP2D6-PGx antipsychotics’ and estimate the proportion of patients eligible for PGx testing based on current pharmacogenomics guidance.

**Methods:**

A cross-sectional study took place extracting data from 243 patients’ medical records to explore psychosis drug prescribing, including drug transitions. Demographic data such as age, sex, ethnicity, and clinical sub-team were collected and summarised. Descriptive statistics explored the proportion of ‘CYP2D6-PGx antipsychotic’ prescribing and the nature of transitions. We used logistic regression analysis to investigate associations between demographic variables and prescription of ‘CYP2D6-PGx antipsychotic’ versus ‘non-CYP2D6-PGx antipsychotic’.

**Results:**

Two-thirds (164) of patients had been prescribed a ‘CYP2D6-PGx antipsychotic’ (aripiprazole, risperidone, haloperidol or zuclopenthixol). Over a fifth (23%) of patients would have met the suggested criteria for PGx testing, following two psychosis drug trials. There were no statistically significant differences between age, sex, or ethnicity in the likelihood of being prescribed a ‘CYP2D6-PGx antipsychotic’.

**Conclusions:**

This study demonstrated high rates of prescribing ‘CYP2D6-PGx-antipsychotics’ in an EIP cohort, providing a rationale for further exploration of how PGx testing can be implemented in EIP services to personalise the prescribing of drugs for psychosis.

## Introduction

Early Intervention in Psychosis (EIP) services support people experiencing first-episode psychosis (FEP) or other psychotic disorders (O’Connell et al., 2021). These patients are often antipsychotic-naïve, and despite being more likely to show therapeutic responses to drugs for psychosis, they have a higher risk of experiencing adverse drug reactions (ADRs) (Lieberman et al., 2013). ADRs, along with other factors, contribute to poor adherence to psychosis drugs (Lally et al., 2017; Semahegn et al., 2020). Adherence rates to drugs for psychosis are estimated to be less than 50% (Haddad et al., 2014; Lacro et al., 2002). Yet, adherence to psychosis drugs during FEP has been shown to improve long-term outcomes, including reduced relapse, hospitalisation and suicide rates (Alvarez-Jimenez et al., 2012; Drake et al., 2020; Fedyszyn et al., 2014; Thompson et al., 2018; Tiihonen et al., 2018). Initial experiences with psychosis drugs are important and if ADRs or poor therapeutic responses occur, negative perceptions can develop and make patients less inclined to adhere to them in the future (Yeisen et al., 2017).

Pharmacogenomics (PGx) studies how genetic variation can influence drug response (Hockings et al., 2020). This is broadly broken down into two categories: genetic variants that influence drug pharmacodynamics and those that alter pharmacokinetic processes (Roden et al., 2011). Over 95% of people possess at least one genetic variant with the potential to influence their response to a medicine (McInnes et al., 2021). These variants can increase the risk of poor therapeutic response or ADRs. Pharmacogenetic (PGx) testing allows for the identification of genetic variants that may alter medication response. By genotyping a patient blood or saliva sample, relevant genetic variants can be determined and utilised when prescribing medicines, as part of a personalised prescribing approach (Royal College of Physicians and British Pharmacological Society, 2022).

PGx influences on psychosis drug response have been established (Arranz et al., 2021; Murphy et al., 2022). At present, the strongest evidence is for pharmacokinetic genes, particularly variants of the CYP2D6 metabolic enzyme (Murphy et al., 2022), which is involved in the breakdown of numerous psychosis drugs (Milosavljevic et al., 2021). Poor or intermediate metabolisers are at risk of drug accumulation and ADRs, while rapid or ultra-rapid metabolisers are at risk of treatment inefficacy due to sub-therapeutic plasma drug levels (Milosavljevic et al., 2021). Pharmacogenomics Knowledgebase (PharmGKB) appraises evidence for given drug–gene pairs, by assigning a level of quality for supporting evidence to grade PGx associations (PharmGKB, 2022). Currently, only psychosis drugs with PGx associations for the CYP2D6 gene have been assigned the highest level of evidence (LoE) and have clinical dosing guidance (see Supplemental Appendix A) (DPWG, 2022; Murphy et al., 2022). These recommendations are included in the Dutch Pharmacogenetics Working Group (DPWG) guidelines (Beunk et al., 2024) and the LoE is graded by PharmGKB. Initial dosage reductions are recommended in poor metabolisers taking aripiprazole, risperidone, haloperidol and zuclopenthixol, while dosage increases are recommended for fast metabolisers taking risperidone, haloperidol and zuclopenthixol (Beunk et al., 2024). Several drugs for psychosis also have PGx information listed in their Food and Drug Administration (FDA) drug labels (Eum et al., 2016; FDA, 2023).

There is evidence that variation in the CYP2D6 genotype, leading to non-normal metaboliser status, causes clinically relevant differences in plasma levels of specific psychosis drugs (Milosavljevic et al., 2021). A systematic review found that poor and intermediate CYP2D6 metabolisers had significantly higher plasma levels of aripiprazole, risperidone and haloperidol compared with normal metabolisers (Milosavljevic et al., 2021), placing them at an increased risk of ADRs (Wannasuphoprasit et al., 2022). Current guidelines suggest considering PGx testing when switching antipsychotics if patients have experienced psychosis drug failure, especially if trialled psychosis drugs have similar metabolism routes (Van Westrhenen et al., 2021b). Studies are ongoing to assess the clinical outcomes of adopting PGx-guided psychosis drug prescribing (Jukic et al., 2022; Pelgrim et al., 2024; Su et al., 2021; Tsermpini et al., 2020). It is hoped these will follow the positive findings from early PGx-guided psychosis drug trials (Fleeman et al., 2011) and trials assessing clinical outcomes from PGx-guided prescribing of drugs for depression (Brown et al., 2022).

Due to poor therapeutic response or ADRs (Ostuzzi et al., 2022), patients frequently switch between drugs for psychosis, introducing risks and an increased likelihood of not continuing the new drug (Essock et al., 2006). The benefits of continuing treatment must be balanced against patient toleration of ADRs (Nash et al., 2021). CYP2D6 genotype can predict plasma drug levels and clinical outcomes for some psychosis drugs (Jukic et al., 2019; Milosavljevic et al., 2021; Wannasuphoprasit et al., 2022). PGx has the potential to help understand why these transitions are required and inform the initial selection and dose of drugs for psychosis.

PGx implementation in mental health settings around the globe has been limited (Lunenburg and Gasse, 2020). In Canada and the United States, PGx use in psychiatry is increasing (Anderson et al., 2020; Haga and Kantor, 2018; Matey et al., 2022; Volpi et al., 2018; Young and MacDougall, 2023), albeit mainly in research or via commercial testing. In Europe, some countries such as the Netherlands and Finland have guidance for the use of PGx in psychiatry (Beunk et al., 2024; Brouwer et al., 2022; Duodecim, 2022a, 2022b). The use of PGx testing in mental health in New Zealand has also been reported (Maggo et al., 2019). In the UK, PGx implementation has been limited to oncology (National Health Service (NHS) England, 2021) and paediatrics (McDermott et al., 2022). The UK’s NHS Genomic Medicine Service (GMS) is tasked with helping to embed PGx across the NHS (Robinson, 2020). The NHS GMS has commissioned a pilot feasibility study enabling GP practices to order PGx tests when prescribing commonly used medicines with PGx associations (Wilkinson and Connelly, 2022). This will build on the results of the ‘PREPARE’ trial, a large multicentre PGx trial in Europe that found PGx-guided prescribing reduces ADRs by 30% (Swen et al., 2023), and help inform PGx implementation in the NHS.

This study aimed to explore the prescribing of drugs for psychosis in an EIP cohort in Bradford, Northern England, to investigate the following:

- The proportion of prescribing psychosis drugs with CYP2D6 PGx associations and whether any groups are more likely to be prescribed one.
- Psychosis drug transitions to estimate the proportion of patients who may benefit from a PGx testing intervention at the point of transition.

This will be the first study of its kind to explore the prescribing of psychosis drugs with CYP2D6 PGx associations in a UK EIP cohort.

## Methods

### Study setting

The study was based on a community EIP service provided by the Bradford District Care NHS Foundation Trust (BDCT), an NHS trust providing secondary mental health care to patients in Bradford. Bradford is a city in the North of England, with surrounding semi-urban district areas. It is the fifth most populated district authority in England and possesses the youngest population of any UK city. It is ethnically diverse with two-thirds of the population describing themselves as ‘White’, and a quarter as ‘Asian’/’Asian British’, with ‘Black’/’Black British’, ‘Mixed’/’Mixed British’ and ‘Other’ ethnic groups accounting for the remaining population (Council, 2023).

### Eligibility criteria, sampling strategy and population

**Figure 1.**
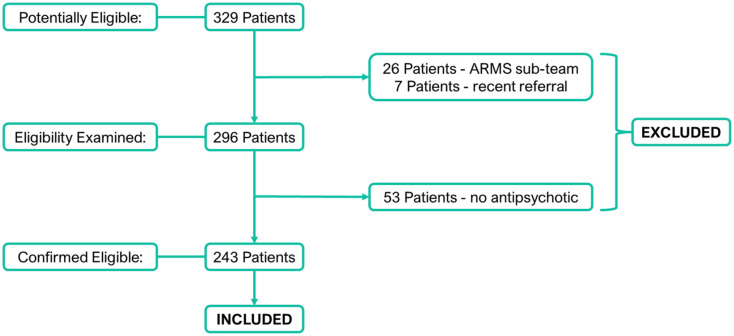
Study inclusion and exclusion flowchart. Flowchart showing patients assessed for eligibility.

Inclusion criteria included being aged 18–65, registered with the BDCT EIP service and being currently prescribed a drug for psychosis. Exclusion criteria included no current psychosis drug prescription, or those not receiving the full EIP service care programme (e.g. those in the ‘At Risk Mental State’ (ARMS) sub-team). All 329 patients registered with the BDCT EIP service on the 15th December 2021 were assessed for eligibility (see Figure 1). In all, 26 patients were initially excluded as they were specifically registered with the ARMS sub-team that specialises in helping people who are deemed to be ‘at risk’ of acquiring the criterion required to receive the full care package, but at present are not believed to meet these criteria and are instead having their clinical progression monitored. A further seven patients were excluded due to a recent referral, meaning that they were yet to have their initial consultation with the service. In total, 53 patients were excluded from the study because they were not prescribed a drug for psychosis at the time of data collection, leaving a total of 243 included patients. No included patients had received PGx testing.

### Data collection methods

The electronic health records (EHR) for each included patient were screened and data were extracted using a data collection tool that was developed using Google Forms. All data collected and stored on Google Forms were pseudonymised by assigning a study ID number to each patient’s data and ensuring only non-identifying data were collected. Therefore, no individual could be identified from the data. The teams within the service were also pseudonymised: each team was assigned a letter, A, B, C or D. The teams within the service cover different geographical locations. Data extracted included demographic data including age, sex, ethnicity and registered sub-team. Antipsychotic history was also extracted, including currently and previously prescribed drugs for psychosis, and if reported the reason for psychosis drug transitions.

### Defining ‘CYP2D6-PGx Antipsychotic’ and ‘non-CYP2D6-PGx Antipsychotic’

**Figure 2.**
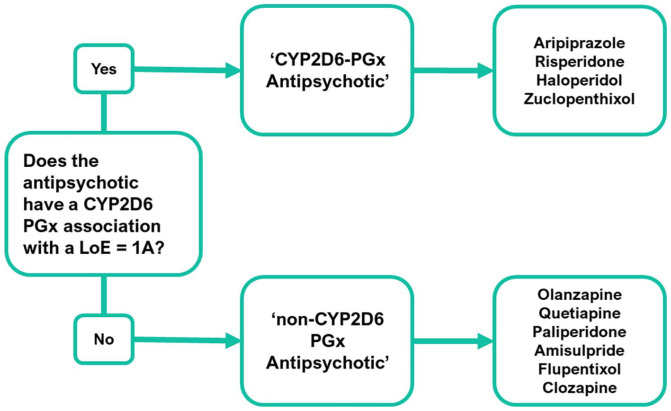
Psychosis drugs classification process. To be classified as a *‘CYP2D6-PGx antipsychotic’* for the study, a psychosis drug required a CYP2D6 PGx association that has a supporting level of evidence (LoE) of the highest possible assigned level 1A (as graded by PharmGKB).

Prior to analysis, drugs for psychosis or antipsychotics were grouped as either a ‘CYP2D6-PGx Antipsychotic’ or a ‘non-CYP2D6-PGx Antipsychotic’ (see Figure 2). Grouping depended on the current PGx guidance, available on the PharmGKB website (date accessed: 1st August 2022); a National Institute for Health funded, open-access database that summarises genetic variants that can influence response to medication (PharmGKB, 2022). PharmGKB gathers, summarises and shares information relating to clinically actionable drug–gene associations or ‘PGx interactions’. They present clinical recommendations from medicines agencies (e.g., the FDA) and pharmacogenetic organisations, such as the Dutch Working Pharmacogenetics Group (DWPG). They also assign an LoE to drug–gene pairs which range from level 4 (unsupported) through to level 1A (high). LoEs are determined using a scoring system that considers if prescribing guidance exists, based on variant-specific clinical guidelines or drug labels, and whether there are independent publications supporting recommendations.

The PharmGKB database was searched for the psychosis drugs prescribed within the BDCT EIP cohort. If a drug for psychosis had an associated CYP2D6 variant listed, with a supporting LoE of level 1A, then it met the criteria to be classified as a ‘CYP2D6-PGx Antipsychotic’; aripiprazole, risperidone, haloperidol and zuclopenthixol all met this criterion. Other drugs for psychosis lacking this were assigned to the ‘non-CYP2D6-PGx Antipsychotic’ group. For specific dosing recommendations for ‘CYP2D6-PGx Antipsychotics’, see Supplemental Appendix A.

### Data analysis

Descriptive statistics were used to analyse the breakdown of the data by sex, age, ethnicity and clinical sub-team, and to describe the proportion of psychosis drug prescribing and the nature of transitions between drugs for psychosis. When analysing the prevalence of prescribing, the unit of analysis was an individual patient. When analysing psychosis drug transitions, the unit of analysis was defined as a switch from one drug for psychosis to another.

A binary logistic regression model was developed using age, sex and ethnicity as predictor variables and the PGx status of currently prescribed psychosis drug as an outcome variable. Adjusted odds ratio (OR) for demographic data was reported with the outcome ‘CYP2D6-PGx Antipsychotic’ (0) versus ‘non-CYP2D6-PGx Antipsychotic’ (1).

Descriptive and inferential statistics were performed using IBM SPSS (Statistical Package for Social Sciences) v27 and Microsoft Excel by Microsoft Corporation. The ‘Strengthening the Reporting of Observational Studies Epidemiology’ guidelines were followed in reporting the results (Elm et al., 2007).

### Ethical process

The study was registered as a service evaluation by the BDCT Research and Development department, as it was considered non-interventional and did not directly impact the care of included patients. No special permission was required to access and collect data, as the intention of the study was to capture the potential burden for PGx testing which could lead to service improvement. The principal researcher (AJ), a clinical pharmacist employed by BDCT, had routine access to the patient EHR from which the data were collected from. Google Forms was used to facilitate the extraction and storage of pseudonymised data only.

## Results

### Prescribed antipsychotics

Table 1 shows that 112 (46%) patients were prescribed a ‘CYP2D6-PGx antipsychotic’, and 131 (54%) patients were prescribed a ‘non-CYP2D6-PGx antipsychotic’, demonstrating a high level of prescribing psychosis drugs with CYP2D6 pharmacogenomic associations.

**Table 1.** Currently prescribed drug for psychosis – ‘CYP2D6-PGx antipsychotic’ versus ‘non-CYP2D6 antipsychotic’.

Characteristic	2D6 PGx (%)	non-2D6 (%)	Total (%)
*N*	112	131	243
Sex			
Female	52 (46.4)	49 (37.4)	101 (41.6)
Male	60 (53.6)	82 (62.6)	142 (58.4)
Age (years)			
17–24	42 (37.5)	47 (35.9)	89 (36.6)
25–30	28 (25.0)	21 (16.0)	49 (20.2)
31–40	22 (19.6)	29 (22.1)	51 (21.0)
41–50	10 (8.9)	22 (16.8)	32 (13.2)
51+	10 (8.9)	12 (9.2)	22 (9.1)
Ethnicity			
White	52 (46.4)	55 (42.0)	107 (44.0)
Asian	49 (43.8)	48 (36.6)	97 (39.9)
Black	4 (3.6)	11 (8.4)	15 (6.2)
Mixed	3 (2.7)	5 (3.8)	8 (3.3)
Other	2 (1.8)	6 (4.6)	8 (3.3)
Not stated	2 (1.8)	6 (4.6)	8 (3.3)
Team			
A	20 (17.9)	12 (9.2)	32 (13.2)
B	36 (32.1)	40 (30.5)	76 (31.3)
C	11 (9.8)	5 (3.8)	16 (6.5)
D	45 (40.2)	74 (56.5)	119 (49.0)

Displayed is the proportion of included patients that are prescribed a ‘CYP2D6-PGx antipsychotic’ in the ‘*2D6 PGx*’ column versus those prescribed a ‘non-CYP2D6-antipsychotic’ in the ‘*non-2D6*’ column, by characteristic (sex, age, ethnicity and team).

There were more males (142, 58%) than females (101, 42%). Patients were mostly young adults; the median age was 29 years (IQR = 17). The cohort was predominantly ‘White’ and ‘Asian’ in ethnicity, with 107 (44%) and 97 (40%) patients, respectively. There were also 15 (6%) ‘Black’ patients, 8 (3%) ‘Mixed’ patients, 8 (3%) patients grouped into the ‘Other’ category and 8 (3%) patients with no ethnicity status recorded. Team ‘D’ was the largest sub-team with 119 (49%) patients.

**Figure 3.**
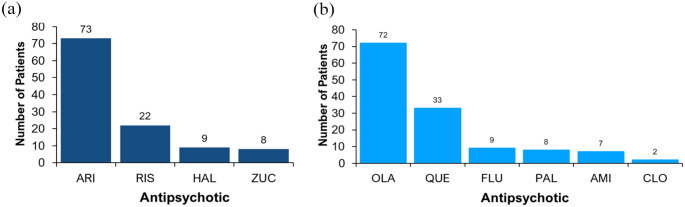
Currently prescribed drugs for psychosis: (a) ‘CYP2D6-PGx antipsychotics’ and (b) ‘non-CYP2D6-PGx antipsychotics’. Currently prescribed psychosis drugs based on PGx grouping. (a) ARI: Aripiprazole; RIS: Risperidone; HAL: Haloperidol; ZUC: Zuclopenthixol. (b) OLA: Olanzapine; QUE: Quetiapine; PAL: Paliperidone; AMI: Amisulpride; FLU: Flupentixol; CLO: Clozapine.

Differences were observed in the demographic characteristics between the ‘CYP2D6-PGx antipsychotic’ group and the ‘non-CYP2D6-PGx antipsychotic’ group, but none of the differences were statistically significant. Figure 3 shows the proportion of prescribing for each drug for psychosis prescribed across the cohort, by antipsychotic grouping. A total of 10 different psychosis drugs were prescribed.

The results of the logistic regression analysis used to investigate the relationship between sex, age and ethnicity and the CYP2D6-PGx status of the psychosis drug prescribed at the time of data collection are shown in Table 2. None of the associations were statistically significant.

**Table 2.** Logistic regression model.

Variable	OR	95%CI		*p*-value
		Lower	Upper	
Age				
Years	1.016	0.992	1.040	0.196
Sex				
Female	1			
Male	1.455	0.856	2.472	0.166
Ethnicity				
White	1			
Asian	0.956	0.542	1.687	0.877
Black	2.433	0.072	8.241	0.153
Mixed	1.850	0.411	8.330	0.423
Other	2.819	0.539	14.750	0.220
Not stated	2.948	0.563	15.436	0.201

This model analysed the relationship between age, sex and ethnicity on the likelihood of a participant being prescribed a drug for psychosis with CYP2D6 PGx association or not (outcome variable: (0) ‘CYP2D6-PGx antipsychotic’; (1) ‘non-CYP2D6-PGx antipsychotic’). Adjusted odds ratio (OR) has been reported which shows the strength of association between the variable and outcome: OR >1 = stronger association between variable and outcome; OR <1 = weaker association between variable and outcome. For age, the OR represents the change in the strength of association for every one age year gained. For sex and ethnicity, the OR shows the strength of association in comparison to the reference category, which for sex is ‘female’ and for ethnicity is ‘White’. The 95% confidence intervals (CI) show the range in estimates for the OR and because the range between lower and upper CI is above and below 1, the results are not statistically significant.

There were non-significant trends towards males being more likely to be prescribed a ‘non-CYP2D6-PGx antipsychotic’, with an OR = 1.46 (95%CI: 0.87; 2.45). Patients in the ‘Black’ ethnicity group had a higher chance of being prescribed a ‘non-CYP2D6-PGx antipsychotic’ compared to participants in the ‘White’ patient group, OR = 2.43 (95%CI: 0.07; 8.24). ‘Asian’ patients were slightly more likely to be prescribed a ‘CYP2D6-PGx antipsychotic’ compared to ‘White’ patients, OR = 0.96 (95%CI: 0.54; 1.69). Considering age, for each year in age gained patients were slightly more likely to be prescribed a ‘non-CYP2D6-PGx antipsychotic’ OR = 1.02 (95%CI: 0.99; 1.04).

### Psychosis drug transitions

Data about previously prescribed drugs for psychosis were also collected, allowing for exploration of ‘antipsychotic-to-antipsychotic’ transitions. A total of 246 transitions took place within the cohort. The median number of transitions occurring per patient was 1 (range = 0–5). The nature of ‘antipsychotic-to-antipsychotic’ transitions was also explored, as some were more prevalent than others. The type of transition was grouped, based on the CYP2D6-PGx status of the initial and new drug for psychosis.

1. ‘2D6 to 2D6’ = ‘CYP2D6-PGx antipsychotic’ ⇨ ‘CYP2D6-PGx antipsychotic’
2. ‘2D6 to non-2D6’‘CYP2D6-PGx antipsychotic’ ⇨ ‘non-CYP2D6-PGx antipsychotic’
3. ‘non-2D6 to 2D6’ = ‘non-CYP2D6-PGx antipsychotic’ ⇨ ‘CYP2D6-PGx antipsychotic’
4. ‘non-2D6 to non-2D6’ = ‘non-CYP2D6-PGx antipsychotic’ ⇨ ‘non-CYP2D6-PGx antipsychotic’

**Figure 4.**
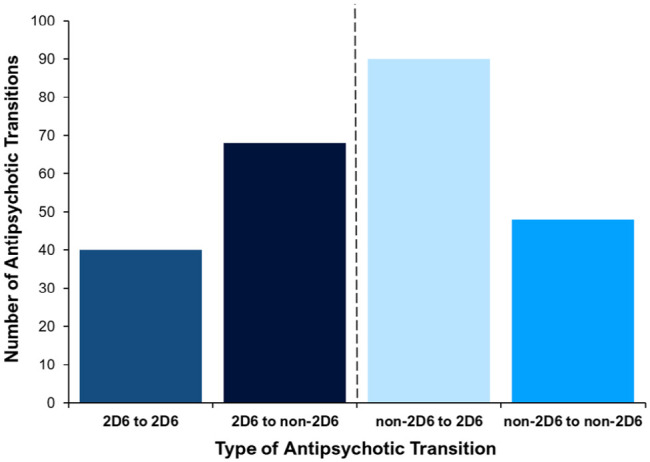
Proportion of retrospective psychosis drug transitions grouped by type of transition. Displayed is the proportion of ‘antipsychotic-to-antipsychotic’ transitions, grouped by the different types of psychosis drug transition. Transition types: ‘*2D6 to 2D6*’ = ‘CYP2D6-PGx antipsychotic’ to another ‘CYP2D6-PGx antipsychotic’; ‘*2D6 to non-2D6*’ = ‘CYP2D6-PGx antipsychotic’ to a ‘non-CYP2D6-PGx antipsychotic’; ‘*non-2D6 to 2D6*’ = ‘non-CYP2D6-PGx antipsychotic’ to a ‘CYP2D6-PGx antipsychotic’; ‘*non-2D6 to non-2D6*’ = ‘non-CYP2D6-PGx antipsychotic’ to ‘non-CYP2D6-PGx antipsychotic’.

Figure 4 shows the proportion of different types of ‘antipsychotic-to-antipsychotic’ transition by group. Of these transitions, 40 (16.3%) were ‘2D6 to 2D6’ transitions, 68 (27.6%) were ‘2D6 to non-2D6’, 90 (36.6%) were ‘non-2D6 to 2D6’ transitions and 48 (19.5%) were ‘non-2D6 to non-2D6’ transitions. This demonstrates a high number of transitions that involve a ‘CYP2D6-PGx antipsychotic’.

Table 3 shows the number of ‘antipsychotic-to-antipsychotic’ transitions by characteristic (sex, age, ethnicity and team). Most patients (105; 43.2%) had 0 transitions, and as the number of transitions per patient increases the number of patients decreases.

**Table 3.** Retrospective psychosis drug transitions.

Characteristics	0 (%)	1 (%)	2 (%)	3 (%)	4+ (%)	Total (%)
*N*	**105 (43.2)**	**63 (25.9)**	**50 (20.6)**	**18 (7.4)**	**7 (2.9)**	**243 (100)**
Sex						
Female	48 (45.7)	27 (42.9)	15 (30)	8 (44.4)	3 (42.9)	101 (41.6)
Male	57 (54.3)	36 (57.1)	35 (70)	10 (55.6)	4 (57.1)	142 (58.4)
Age (years)						
17–24	35 (33.3)	23 (36.5)	22 (44)	6 (33.3)	3 (42.9)	89 (36.6)
25–30	22 (21)	13 (20.6)	10 (20)	2 (11.1)	2 (28.6)	49 (20.2)
31–40	22 (21)	14 (22.2)	8 (16)	7 (38.9)	0 (0)	51 (21)
41–50	16 (15.2)	7 (11.1)	6 (12)	2 (11.1)	1 (14.3)	32 (13.2)
51+	10 (9.5)	6 (9.5)	4 (8)	1 (5.6)	1 (14.3)	22 (9.1)
Ethnicity						
White	43 (41)	27 (42.9)	22 (44)	12 (66.7)	3 (42.9)	107 (44)
Asian	38 (36.2)	31 (49.2)	20 (40)	5 (27.8)	3 (42.9)	97 (39.9)
Black	8 (7.6)	2 (3.2)	3 (6)	1 (5.6)	1 (14.3)	15 (6.2)
Mixed	7 (6.7)	1 (1.6)	0 (0)	0 (0)	0 (0)	8 (3.3)
Other	4 (3.8)	2 (3.2)	2 (4)	0 (0)	0 (0)	8 (3.3)
Not stated	5 (4.8)	0 (0)	3 (6)	0 (0)	0 (0)	8 (3.3)
Team						
1	15 (14.3)	9 (14.3)	5 (10)	2 (11.1)	1 (14.3)	32 (13.2.)
2	32 (30.5)	17 (27)	16 (32)	8 (44.4)	3 (42.9)	76 (31.3)
3	8 (7.6)	6 (9.5)	2 (4)	0 (0)	0 (0)	16 (6.6)
4	50 (47.6)	31 (49.2)	27 (54)	8 (44.4)	3 (42.9)	119 (49)

Displayed in the columns are the *number of ‘antipsychotic-to-antipsychotic’ transitions*, ranging from 0 transitions (i.e. those still prescribed the same initial drug for psychosis) to 4+ transitions. Each row shows the proportion of each number of transitions by characteristic *sex, age, ethnicity* and *team.*

**Figure 5.**
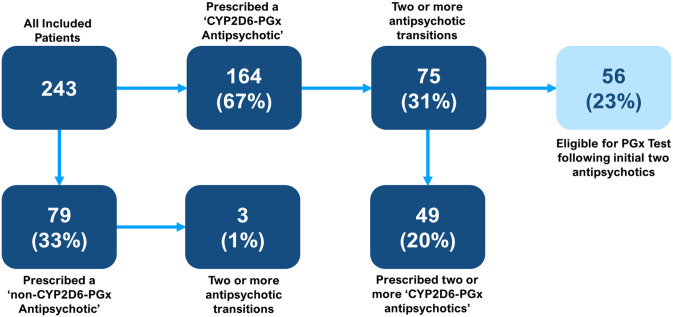
Eligibility for PGx testing. Flow diagram showing the likelihood of included patients being prescribed a ‘CYP2D6-PGx antipsychotic’ at any time (either currently or previously prescribed) and of the proportion that experienced two or more psychosis drug transitions. More specifically, the flow diagram shows that of those who experienced two or more transitions, 49 (20%) patients were prescribed two or more ‘CYP2D6-PGx antipsychotics’ and 56 (23%) patients met the suggested eligibility for having a PGx test, based on current guidelines (Van Westrhenen et al., 2021b).

When exploring retrospective transitions, it was found that 164 (67%) patients had been prescribed a ‘CYP2D6-PGx antipsychotic’ at any time, versus 79 (33%) patients who had never been prescribed a ‘CYP2D6-PGx antipsychotic’. Of those prescribed a ‘CYP2D6-PGx antipsychotic’, it was observed that 75 (31%) patients had two or more transitions and that of these patients 56 (23%) would have met the criteria to offer a PGx test, based on current guidance (Van Westrhenen et al., 2021a), following the first two psychosis drug trials. In comparison, of the 79 (33%) patients prescribed a ‘non-CYP2D6-PGx antipsychotic’, only 3 (1%) patients had two or more transitions. Of all included patients, 49 (20%) trialled two or more ‘CYP2D6 PGx antipsychotics’. A summary of eligibility for PGx testing is found in Figure 5.

**Figure 6.**
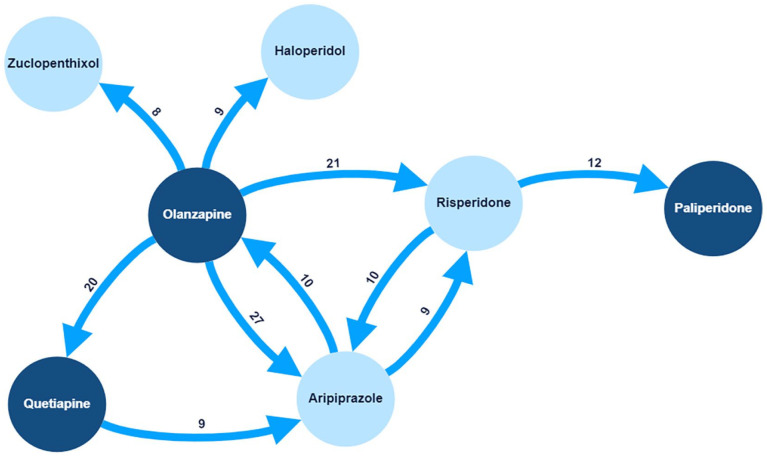
Common psychosis drug transitions. Mapped are the 10 *most prevalent psychosis drug transitions* that occurred for included patients. Highlighted in *light blue* are *‘CYP2D6-PGx antipsychotics’.* Only one transition (*olanzapine to quetiapine*) did not involve a ‘CYP2D6-PGx antipsychotic’. The number assigned to each arrow is the total count for that specific transition (out of a total of 246 transitions).

**Figure 7.**
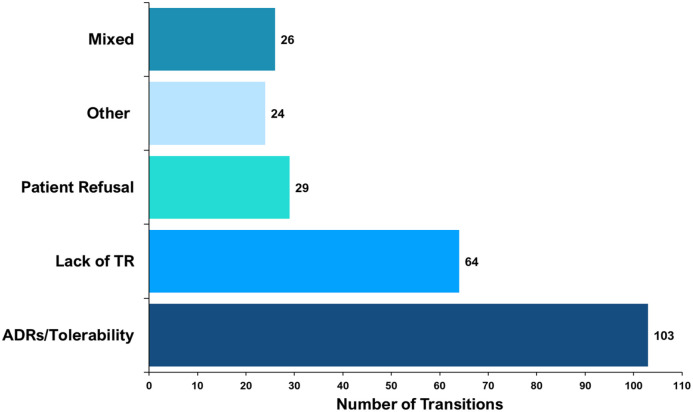
Documented reason(s) for psychosis drug transitions. ADRs/Tolerability was the most common reason, followed by a lack of therapeutic response (lack of TR), and then patient refusal to take and mixed/more than one documented reason. Other reasons included a prolonged period in symptom remission, a switch to an intramuscular depot formulation and when the reason was not stated.

The proportion of specific ‘antipsychotic-to-antipsychotic’ transitions differed. Olanzapine to aripiprazole was the most common transition, occurring 27 times (out of 246 transitions). Figure 6 shows the 10 most prevalent specific transitions by weight. Of these, only the olanzapine to quetiapine transition did not involve a ‘CYP2D6-PGx antipsychotic’. When recorded, data about the reason(s) for ‘antipsychotic-to-antipsychotic’ transitions were extracted. Contributing factors varied by specific psychosis drug. Of the 246 transitions, the most common documented reason was ADRs/tolerability 103 (41.9%), followed by a lack of therapeutic response 64 (26%). Figure 7 shows the documented reasons for transitions.

**Figure 8.**
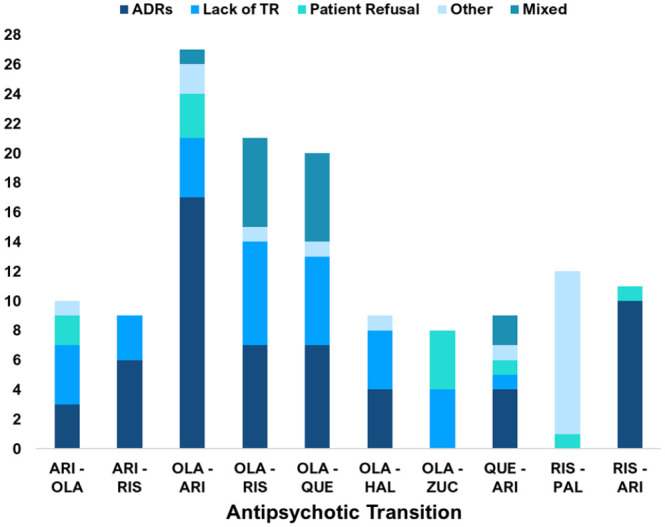
Common psychosis drug transitions stratified by documented reason (s) for the transition. Displayed are the *documented reasons* for ‘antipsychotic-to-antipsychotic’ transitions for *each of the top ten most common individual-specific transition types*. ADRs/Tolerability issues were the most common reasons for these transitions, followed by a Lack of Therapeutic Response (Lack of TR) and then ‘Other’ reasons. Although the proportion of reasons varied by the specific antipsychotic-to-antipsychotic transition. For the ‘Risperidone to Paliperidone’ switch, all the ‘Other’ reasons were due to a switch from oral Risperidone to a Paliperidone intramuscular depot injection. Less common reasons included Patient Refusal to Take and ‘Mixed’ reasons. ARI: aripiprazole; RIS: risperidone; HAL: haloperidol; ZUC: zuclopenthixol; OLA: olanzapine; QUE: quetiapine; PAL: paliperidone.

The reasons for the ten most common transitions also varied (see Figure 8), 57 (42%) were due to ADRs or tolerability issues, 33 (24%) were due to a lack of therapeutic response and 15 (11%) were due to ‘Mixed’ reasons. The 18 (13%) ‘Other’ reasons included a prolonged period in symptom remission, switching to an intramuscular depot formulation and when the reason was not stated. For the ‘Risperidone to Paliperidone’ transition, ‘Other’ reasons accounted for 11 out of the 12 transitions, all of which were due to switching to an intramuscular depot injection.

## Discussion

This study has for the first time demonstrated high rates of prescribing drugs for psychosis with dosing recommendations based on CYP2D6 genotype in an EIP cohort, yet adoption of PGx testing to determine CYP2D6 genotype is to date limited. Some 46% of patients were prescribed a ‘CYP2D6-PGx antipsychotic’ at the time of data collection and 66% had been prescribed one at any time. PGx testing is a reliable method to determine the CYP2D6 genotype, which can be used to guide psychosis drug prescribing and may improve treatment outcomes (Van Westrhenen et al., 2020). Most psychosis drug transitions (80%) in this cohort involved a ‘CYP2D6-PGx antipsychotic’. Based on current guidance (Van Westrhenen et al., 2021b), nearly one in four (23%) included patients would have been eligible for a PGx test after two psychosis drug trials.

A regression model demonstrated that age, sex and ethnicity are poor predictors of whether patients were prescribed a ‘CYP2D6-PGx antipsychotic’, suggesting there are no specific groups that CYP2D6 PGx testing should be prioritised. These study findings highlight that prescribing in EIP cohorts should be a priority area for pharmacogenomic testing roll out, due to the high proportion of prescribing ‘CYP2D6-PGx antipsychotics’. We propose that a tailored PGx-guided prescribing approach should be equally available to patients in EIP services, to guide ‘CYP2D6-PGx antipsychotic’ prescribing.

A lack of therapeutic response (26%) and ADRs (42%) to psychosis drugs were the most common reasons for transitions, further demonstrating a currently unmet clinical need for many patients (Guinart et al., 2022). An individual’s genetics can influence these treatment outcomes (Murphy et al., 2022). Up to a third of people may have a non-normal (poor, intermediate or rapid) metaboliser phenotype for the CYP2D6 gene (Gaedigk et al., 2017), increasing their risk of ADRs or inefficacy when prescribed medicines metabolised by CYP2D6 (Fleeman et al., 2011; Jukic et al., 2019; Milosavljevic et al., 2021; Wannasuphoprasit et al., 2022). We found that 44% of transitions were from a ‘CYP2D6-PGx antipsychotic’ and importantly those prescribed a ‘CYP2D6-PGx antipsychotic’ were more likely to have two or more transitions. Adopting PGx testing in EIP services would allow for exploration of when CYP2D6 genotype has contributed to psychosis drug failure because of ADRs or inefficacy associated with a ‘CYP2D6-PGx antipsychotic’ and enable personalised prescribing of drugs for psychosis to reduce the risk of poor treatment outcomes.

Poor adherence to psychosis drugs is a potential contributing factor in transitions, and ‘patient refusal to take’ accounted for 12% of observed transitions, capturing when clinicians were aware their patient would not take their prescribed drug for psychosis. This does not explain the common issue of poor or non-adherence to psychosis drugs (Haddad et al., 2014; Lacro et al., 2002) and this will likely have influenced the results, particularly the documented reasons for transitions. A lack of therapeutic response was the second most documented reason for switching psychosis drugs, and it is also probable that poor or non-adherence contributed to a proportion of those with a lack of therapeutic response documented as the reason they switched drugs for psychosis.

Psychosis drug dose and duration could have also influenced transitions, including the documented reasons for switches. Non-optimal dosing, either too low or too high, could have contributed to instances when a lack of therapeutic response or side effects were documented as reasons for psychosis drug transitions. Similarly, when side effects were documented as the reason for the transition, the length of treatment was not accounted for, yet those on treatment for longer are more likely to experience side effects (Burschinski et al., 2023; Correll et al., 2018).

Unsurprisingly, more than half (138, 57%) of the patients had one or more psychosis drug transitions. When due to safety concerns, switching is valid and necessary; however, clinical evidence to support switching drugs for psychosis to improve symptom response is lacking (Samara et al., 2018). Yet, clinicians often rely on switches, attempting to improve clinical outcomes (Barnes et al., 2020). Partly due to similar efficacy levels, there is a lack of guidance to help choose between psychosis drugs (Huhn et al., 2019), and clinicians may often rely on their clinical judgement and make prescribing decisions based on subjective experience or preference for certain psychosis drugs (Bhugra et al., 2011; Fava, 2013; Rajendran et al., 2012). PGx offers a more evidence-based approach to selecting and dosing drugs for psychosis, by providing additional clinical information to help inform prescribing decisions. This may reduce the need for transitions or help inform prescribing when switches are necessary, by personalising the initial selection and dosing of psychosis drugs to individuals, to improve tolerability and likelihood of therapeutic response.

Aripiprazole (30%) and olanzapine (29.6%) were the most commonly prescribed drugs for psychosis, representing similar prescribing rates to those previously reported in EIP services (Fallon et al., 2021). Multiple information sources recommend aripiprazole dosing should be tailored to CYP2D6 metaboliser status (Commitee, 2022; DPWG, 2022; FDA, 2014). A previous study demonstrated that the CYP2D6 genotype significantly affects plasma levels of aripiprazole and risperidone and that for risperidone there was a significant effect of the CYP2D6 genotype on therapeutic failure (Jukic et al., 2019). Over a third (39.1%) of patients in our study were prescribed aripiprazole or risperidone. PGx testing is required so that aripiprazole and risperidone prescribing can be tailored to the genetic profile of individuals. EIP services would be ideal for integrating pharmacogenomics, due to the strong evidence supporting the role of PGx in aripiprazole and risperidone response, and the high use of these antipsychotics in this setting, as demonstrated by this study.

The current focus of psychiatry pharmacogenomics centres on pharmacokinetic processes (Van Westrhenen et al., 2021b). As evidence emerges, clinical recommendations based on pharmacodynamic pharmacogenes may be developed, and for example, could be used to identify patients with pharmacogenetic risk factors for antipsychotic-induced weight gain (AIWG). AIWG is a common ADR, particularly with olanzapine which was the most transitioned from psychosis drug in this study. A meta-analysis found a range of genes were significantly associated with AIWG (Zhang et al., 2016), many of which encoded psychosis drug pharmacodynamic targets. Regarding pharmacokinetic genes, a meta-analysis found that reduced CYP2D6 function is also associated with AIWG (Wannasuphoprasit et al., 2022). AIWG increases the risk of cardiovascular disease and leads to poorer long-term physical health outcomes (Dayabandara et al., 2017). Research is required to explore if a PGx-guided prescribing approach is effective in identifying those at risk of AIWG. EIP services are an ideal environment to explore this, during early psychosis drug therapy prior to downstream negative effects of AIWG.

Although pharmacogenomics offers a new approach to predict psychosis drug response and inform prescribing (Murphy et al., 2022; Royal College of Physicians and British Pharmacological Society, 2022), therapeutic drug monitoring (TDM) is a traditional method to monitor psychosis drug plasma levels and personalise dosing (Patteet et al., 2012). However, TDM is reactive and can only determine plasma levels and explain response once a drug for psychosis has been initiated and is often only used when inefficacy or side effects have occurred, to help inform clinician assessment and decision-making. By contrast, PGx testing offers a proactive approach, capable of predicting psychosis drug response before initiation or during challenging psychosis drug transitions. A combination of PGx testing prior to prescribing and TDM throughout psychosis drug therapy could have potential synergistic benefits (Cuvelier et al., 2023).

There are several limitations to the study findings. Due to time constraints and limited resources, genotyping to determine CYP2D6 gene variants among study participants was not performed. This would have allowed for further exploration of the impact that CYP2D6 genotype has on the outcomes from being prescribed a ‘CYP2D6-PGx antipsychotic’ versus a ‘non-CYP2D6-PGx antipsychotic’.

Other factors can also affect metabolism, including phenoconversion, a concept that explains the impact on CYP2D6 enzyme function caused by concomitantly prescribed medicines that inhibit or induce CYP2D6 resulting in a clinical phenotype that is different from the phenotype expected based on genotyping (Cicali et al., 2021). Such drug–drug interactions can lead to fluctuations in drug plasma levels (Prior and Baker, 2003). The study only explored one route of psychosis drug metabolism using CYP2D6, and despite it playing a major role in metabolism, other cytochrome P450 enzymes, such as CYP1A2, CYP2C9/19 or CYP3A4/5 offer alternate metabolism routes for psychosis drugs (Ravyn et al., 2013). Aripiprazole, risperidone and haloperidol are metabolised by CYP2D6 but can also use CYP3A4 for metabolism (Fang et al., 1999; Ravyn et al., 2013). Future clinical pharmacogenomics studies should collect data on co-medications, to explore the impact of phenoconversion on the importance of PGx test results.

Limitations in the data collection tool and data collected also exist. Clarity in documenting clinical outcomes, such as ADRs and symptom response, and the reasons for psychosis drug transitions within the patient’s EHR varied. Some documentation was comprehensive providing clear justifications for antipsychotic transitions backed up by side effects or symptom scores, while in many instances, this detail was lacking in the EHR. Commonly documentation about ADRs was vague and non-specific. Similarly, the reporting of symptom outcomes was often unclear and did not stipulate symptom scores. Due to this inconsistency and lack of detail in documentation, the data collection tool did not capture clinical outcomes such as symptom or side effect scores. These challenges highlight a need for better recording of ADRs and symptom scores, and improved tools to standardise EHR documentation.

Furthermore, there were limitations in how the antipsychotic grouping took place. Only psychosis drugs assigned a LoE = Level-1A for dosing guidance based on CYP2D6 genotype were included in the ‘CYP2D6-PGx’ group. This means that some drugs for psychosis with less strongly evidenced PGx associations were excluded from the PGx group because they lacked the highest LoE. The study focused on the PGx associations with the current highest LoE, as these are the most evidenced genes to offer PGx testing for. This may change as more evidence emerges and further psychosis drugs could have clinical recommendations that are associated with robust evidence.

Finally, a potential confounding factor in the results was the impact of a clinician’s personal preference when selecting psychosis drugs to prescribe. Due to the lack of difference in efficacy between psychosis drugs (Huhn et al., 2019; Leucht et al., 2023), and the lack of robust pathways to help selection, clinicians often rely on their clinical judgement and previous experience when prescribing drugs for psychosis (Bhugra et al., 2011; Fava, 2013). This may have influenced the patterns of psychosis drug prescribing observed if clinicians had a preference to prescribe certain drugs for psychosis.

More robust evidence supporting the clinical utility and cost-effectiveness of PGx-guided psychosis drug prescribing is required. Future research should also address implementation issues hindering the adoption of pharmacogenomics in clinical practice (Jameson et al., 2021; Pirmohamed, 2023). Issues such as training and education about pharmacogenomics must be addressed, especially given that access to PGx testing is increasing (Anderson et al., 2020; Rendell et al., 2022; Volpi et al., 2018). This will ensure that healthcare professionals, including pharmacists, possess the necessary skills and knowledge to benefit from the successful adoption of pharmacogenomics. In the UK, the NHS GMS will be key to providing this (Robinson, 2020). A global pharmacogenomics approach can ensure widespread successful uptake of PGx testing (Chenoweth et al., 2020). Further research is required to explore how services can embed pharmacogenomics into clinical pathways and perspectives towards pharmacogenomics. How PGx testing is communicated with patients, in context-specific services, is a key factor to be investigated too.

## Conclusion

This study is the first to highlight high rates of prescribing drugs for psychosis with clinical recommendations based on the CYP2D6 genotype in EIP services, yet PGx testing to determine the CYP2D6 genotype is not available in EIP settings. It has shown that many psychosis drug transitions involve a ‘CYP2D6-PGx antipsychotic’ and some transitions were directly between these psychosis drugs. Such transitions are a key area where PGx interventions may be of benefit, by acting as a tool to inform prescribing decisions in clinically challenging situations. ADRs and poor therapeutic response were common factors contributing to psychosis drug therapy failure, both of which may be addressed using PGx to personalise prescribing to achieve safer and more effective treatment outcomes. Further pharmacogenomics research is required, from discovering new genetic variants to incorporation of PGx into clinical trials. The impact of performing CYP2D6 PGx testing on psychosis clinical outcomes should also be investigated. Regarding PGx implementation in clinical practice, future research should aim to explore views towards using PGx to aid prescribing in specific patient populations, such as those in EIP services.

## Supplemental Material

sj-docx-1-jop-10.1177_02698811241238283 – Supplemental material for Proportion of Antipsychotics with CYP2D6 Pharmacogenetic (PGx) Associations Prescribed in an Early Intervention in Psychosis (EIP) Cohort: A Cross-Sectional Study Supplemental material, sj-docx-1-jop-10.1177_02698811241238283 for Proportion of Antipsychotics with CYP2D6 Pharmacogenetic (PGx) Associations Prescribed in an Early Intervention in Psychosis (EIP) Cohort: A Cross-Sectional Study by Adam Jameson, Muhammad Faisal, Beth Fylan, Greg C Bristow, Jaspreet Sohal, Caroline Dalton, Gurdeep S Sagoo, Alastair G Cardno and Samantha L McLean in Journal of Psychopharmacology
