# Supplementary material for: Proportion of Antipsychotics with CYP2D6 Pharmacogenetic (PGx) Associations Prescribed in an Early Intervention in Psychosis (EIP) Cohort: A Cross-Sectional Study
Source: J Psychopharmacol. 2024 Mar 17;38(4):382–94. doi: 10.1177/02698811241238283 (PMC11010551; doi:10.1177/02698811241238283)
Supplement: sj-docx-1-jop-10.1177_02698811241238283 – Supplemental material for Proportion of Antipsychotics with CYP2D6 Pharmacogenetic (PGx) Associations Prescribed in an Early Intervention in Psychosis (EIP) Cohort: A Cross-Sectional Study [file sj-docx-1-jop-10.1177_02698811241238283.docx]

## **Appendix A:**

| **Psychosis Drugs with PGx guidance from DWPG based on CYP2D6 genotype *(18, 20-22)*.** | | |
| --- | --- | --- |
| **Psychosis Drug** | **Level of Evidence** | **Clinical Recommendation** |
| Aripiprazole | 1A | *CYP2D6 PM:*  Maximum dose of 10mg/day or 300mg/month. |
| Risperidone | 1A | *CYP2D6 PM:*  Use 67% of the standard dose. If CNS side effects are problematic reduce further to 50% of the standard dose. |
|  |  | *CYP2D6 UM:*  Choose an alternative antipsychotic  OR titrate the risperidone dose based on the maximum dose for paliperidone –  Maximum dose PO paliperidone 12mg/day = 6mg/day PO risperidone. |
| Haloperidol | 1A | *CYP2D6 PM:*  Use 60% standard dose. |
|  |  | *CYP2D6 UM:*  Use 150% of the standard dose  OR choose an alternative antipsychotic |
| Zuclopenthixol | 1A | *CYP2D6 PM:*  Use 50% of the standard dose |
|  |  | *CYP2D6 IM:* Use 75% of the standard dose |
|  |  | *CYP2D6 UM:*  Insufficient evidence to make a dosing recommendation however if effectiveness is ineffective try an increased dose but do not exceed 150% of the standard dose. |

***Description:*** *aripiprazole, risperidone, haloperidol and zuclopenthixol are at present the only drugs for psychosis with* ***clinical recommendations*** *from* ***DWPG guidelines*** *based on CYP2D6 genotype, with the highest available* ***level of evidence*** *grading from* ***PharmGKB.
PM =*** *Poor Metaboliser;* ***IM*** *= Intermediate Metaboliser;* ***UM*** *= Ultra-Rapid Metaboliser*
